# Late presentation increases risk and costs of non-infectious comorbidities in people with HIV: an Italian cost impact study

**DOI:** 10.1186/s12981-016-0129-4

**Published:** 2017-02-16

**Authors:** Giovanni Guaraldi, Stefano Zona, Marianna Menozzi, Thomas D. Brothers, Federica Carli, Chiara Stentarelli, Giovanni Dolci, Antonella Santoro, Ana Rita Domingues Da Silva, Elisa Rossi, Julian Falutz, Cristina Mussini

**Affiliations:** 1 Department of Medical and Surgical Sciences for Adults and Children, Clinic of Infectious Diseases, University of Modena and Reggio Emilia, Emilia-Romagna, Modena, Italy; 2 Faculty of Medicine, Dalhousie University, Halifax, NS Canada; 3 McGill University Health Center, Montreal, QC Canada; 4 Infectious Disease Department, Hospital Beatriz Ângelo, Regiao de Lisboa, Loures, Portugal; 5 Health Care Department, Cineca-Interuniversity Consortium, Casalecchio di Reno, Emilia-Romagna, Bologna, Italy

**Keywords:** HIV, Late presentation, Comorbidities, Costs

## Abstract

**Background:**

Late presentation (LP) at the time of HIV diagnosis is defined as presentation with AIDS whatever the CD4 cell count or with CD4 <350 cells/mm. The objective of our study was to assess the prevalence of non-infectious comorbidities (NICM) and multimorbidity among HIV-positive individuals with and without a history of LP (HIV + LP and HIV + EP, respectively), and compare them to matched HIV-negative control participants from a community-based cohort. The secondary objective was to provide estimates and determinants of direct cost of medical care in HIV patients.

**Methods:**

We performed a matched cohort study including HIV + LP and HIV + EP among people attending the Modena HIV Metabolic Clinic (MHMC) in 2014. HIV-positive participants were matched in a 1:3 ratio with HIV-negative participants from the CINECA ARNO database. Multimorbidity was defined as the concurrent presence of ≥2 NICM. Logistic regression models were constructed to evaluate associated predictors of NICM and multimorbidity.

**Results:**

We analyzed 452 HIV + LP and 73 HIV + EP participants in comparison to 1575 HIV-negative controls. The mean age was 46 ± 9 years, 27.5% were women. Prevalence of NICM and multimorbidity were fourfold higher in the HIV + LP compared to the general population (p < 0.001), while HIV + EP present an intermediate risk. LP was associated with increased total costs in all age strata, but appear particularly relevant in patients above 50 years of age, after adjusting for age, multimorbidity, and antiretroviral costs.

**Conclusions:**

LP with HIV infection is still very frequent in Italy, is associated with higher prevalence of NICM and multimorbidity, and contributes to higher total care costs. Encouraging early testing and access to care is still urgently needed.

**Electronic supplementary material:**

The online version of this article (doi:10.1186/s12981-016-0129-4) contains supplementary material, which is available to authorized users.

## Background

The World Health Organization defines “late presentation” (LP) as a new HIV diagnosis with concurrent acquired immunodeficiency syndrome (AIDS) defining events, whatever the CD4+ T cell count, or else a new human immunodeficiency virus (HIV) diagnosis with CD4+ count less than 350 cells/mm^3^ [1, 2]. LP is associated with adverse outcomes, including shorter life expectancy [3–5], and may also impair the efficacy and tolerability of combined antiretroviral therapy (cART) [4, 6]. Although most HIV-positive individuals who begin therapy with a CD4+ count above 200 cells/mm^3^ eventually achieve a CD4+ count >500 cells/mm^3^, those with LP are far less likely to ever experience a robust CD4+ cell gain [7]. LP has also been associated with enhanced risk for drug toxicities including anemia [8] and immune reconstitution inflammatory syndrome (IRIS) [9]. Approximately 40–60% of new HIV diagnoses in Italy are made in the context of LP [10]. The Collaboration of Observational HIV Epidemiological Research Europe (COHERE) study observed a small decrease in LP across Europe from 57.3% in 2000 to 51.7% in 2010/11, suggesting that the prevalence of LP remains stubbornly high over time [5].

Older age represents a consistent risk factor for LP [11–14], as a result of barriers to HIV testing among older adults at the individual, medical and public health policy levels [15, 16]. With an increasing number of people being diagnosed with HIV at an older age, the association between older age and LP is becoming even more significant and it is important to understand how LP might affect the health of people aging with HIV.

Nevertheless, it is still unknown whether there is an association between LP and age-related non-infectious co-morbidities (NICM) among people living with HIV, including cardiovascular disease (CVD), hypertension, type 2 diabetes mellitus (T2DM), chronic kidney disease (CKD), and osteopenia/osteoporosis. These heterogeneous comorbidities recognize age and HIV disease as independent risk factors, and tend to aggregate into complex multimorbidity (MM) patterns (usually defined as two or more NICM being present in the same individual at the same assessment).

The objective of our study was to assess the prevalence of NICM and MM among HIV-positive individuals with and without a history of LP, and compare them to matched HIV-negative control participants from a community-based cohort. The secondary objective was to provide estimates and determinants of direct cost of medical care in LP HIV patients.

## Methods

The study took place at the multidisciplinary Modena HIV Metabolic Clinic (MHMC), which was initiated in 2004 to assess longitudinal metabolic changes among people with HIV. As previously described [17, 18], patients undergo annual comprehensive assessments in multiple domains, including metabolic and endocrinological variables, bone mineral density (BMD), organ function, and social factors.

In the past 10 years a gradual decline in LP was observed in people attending MHMC, similar to that found in other European countries [5, 14, 19]. In 2004 LP prevalence in our cohort was 87%, while in 2012 it was 65% of the new patients.

The current cross-sectional study was performed using data from two groups of individuals with visits to the MHMC in 2014. One group were all patients identified with a history of LP at the time of HIV diagnosis (HIV + LP). The other were all patients without a history of LP, and were therefore early presenters (HIV + EP). LP was defined according to the WHO definition, as a new HIV diagnosis with concurrent AIDS defining events, whatever the CD4+ T-cell count, or else a new HIV diagnosis with CD4+ count less than 350 cells/mm^3^ [1, 2].

HIV + LP and HIV + EP participants were each matched in a 1:3 ratio, on age, sex, and race (all Caucasian) and geographical area of origin, to participants in a community-based cohort of the general population, the CINECA ARNO database [18]. The ARNO Observatory is an on-line, multi-centre observational database in which population-based data is collected and epidemiological methods [20] are used to combine and aggregate large volumes of health and healthcare-related data for each individual patient. These data include primary care provider-generated medication prescriptions, inpatient hospital records and discharge, summaries, diagnostic laboratory tests and radiographic examinations. This information is linked to other sources of patient data (including vital statistics and patient demographics) in order to provide comprehensive tracking of clinical diagnoses and healthcare use trends throughout Italy. Lifestyle, anthropometric and metabolic data are not collected in the CINECA ARNO database. All CINECA ARNO participants included in this study did not have a diagnosis of HIV infection, and were therefore assumed to be HIV negative.

### Outcomes

NICM diagnoses were based according the following criteria previously used in our studies [18]. The category of CVD included the following diagnoses: myocardial infarction, coronary artery disease, peripheral vascular disease, stroke, angina pectoris, coronary artery bypass grafting, and angioplasty. Among MHMC participants, diagnostic criteria for hypertension, T2DM, and CKD included, respectively, blood pressure measurements >140/90 mmHg over two consecutive measurements, fasting serum glucose levels >126 mg/dL and eGFR <60 ml/min using the modification of diet in renal disease (MDRD) estimating equation [21]. Drug tracing criteria used to establish hypertension and T2DM diagnoses in both cohorts included current use of antihypertensive and hypoglycemic drugs. In the HIV-positive groups only we analyzed low bone mineral density (t-score <−2SD) using dual-energy X-ray absorptiometry (DXA).

### Direct healthcare cost assessments

The cost of care analysis is referred to the economic point of view of the Italian public healthcare system, which offers drugs and health assistance free of charge to any HIV-positive patients. Direct healthcare costs were retrospectively analyzed in the calendar year 2013, as previously described [22] using the following indicators:

1. Hospital costs
  Hospitalization costs were calculated by collecting International Classification of Diseases, ninth edition (ICD9) codes in both cohorts. All hospitalization with a primary or secondary ICD-9 diagnosis codes including CVD, hypertension, T2DM, bone fractures, and renal failure diagnosis were selected [23] (Additional file 1: Table S1).
2. Outpatient HIV medical care cost
  With the movement of HIV care into the outpatient setting, it is not possible to evaluate the total cost of medical care using ICD9 codes. For outpatient cares we attributed a mean patient care cost obtained by the most recent reference value, which was able to better define HIV related care cost in patient with cART. This estimate is adjusted for current CD4 count strata, accounting 1381 USD/year for patients with CD4 <75 cell/mmc, 1241 USD/year for 76 < CD4 < 200 cell/mmc and 190 USD/year for CD4 > 201 cell/mmc [24] (Additional file 1: Table S1).
3. NICM medication costs
  NICM medication costs were calculated using pharmaceutical tracing by average wholesale prices obtained by National Pharmaceutical Prontuary of Agenzia Italiana del Farmaco (AIFA) [25].
4. cART medication costs
  cART medication cost were calculated using pharmaceutical tracing, by average hospital-sale price.

We converted hospital cost and outpatient HIV medical care cost from USDs to euros using conversion rate at the 31th of December, 2014 [26]. Total cost was defined as the sum of mean of NICM-costs plus mean of HIV-hospitalization costs plus mean of drug costs.

### Statistical analysis

Comparisons among groups were performed using χ^2^ test for categorical variables with Bonferroni adjusted post hoc analyses (significance level fixed at p < 0.017) and T-test or Mann–Whitney U-test for normally and non-normally distributed continuous variables, respectively. The probability of MM was drawn in the three comparative groups across age distribution using a logistic model. Univariate and multivariable logistic regression models were constructed to determine factors associated with LP using general population as reference after correction for sex and age (in years).

Considering the non-normal distribution of total costs, generalized linear models were constructed to evaluate independent factors associated with total cost in the whole population and in the subgroup of HIV-infected patients, using inverse Gaussian family distribution.

The variables for regression analyses were chosen on the basis of their clinical relevance.

Statistical analyses were performed STATA Software package, Intercooled version 13 for Mac (StataCorp ltd, Collage Station, TX, USA).

## Results

525 individuals presented to the MHMC with new HIV diagnosis in 2014: 452 (85%) were HIV + LP and 73 (15%) were HIV + EP. These HIV-positive MHMC participants were matched to 1575 HIV-negative CINECA ARNO participants. Overall mean age was 46 ± 9 years and 27.5% were women, being the same across study groups per matching criteria.

With regards to HIV patients all the HIV + LP were on ART regimen, while 30.3% of HIV + EP had not yet started ARV.

Table 1 shows descriptive characteristics of the sample and their association with HIV + LP in comparison to HIV + EP.

**Table 1:** Demographic, anthropometric and metabolic characteristics associated with late presentation

	All HIV + Patients (n = 525)	HIV + LP (n = 452)	HIV + EP (n = 73)	OR (95% CI)	p value
Men (n)	381 (72.5%)	319 (71.2%)	60 (82.2%)	0.59 (0.28–1.00)	0.050
Age (mean, SD)	46 (±9)	46 (±8)	43 (±10)	1.03 (1.01–1.08)	0.010
Current CD4 (n/microL) (median, IQR)	590 (441–756)	570 (423–729)	750 (589–981)	0.997 (0.996–0.998)	<0.001
Smoke					
None	318 (60.6%)	282 (62.4%)	36 (49.3%)	1 (ref.)	–
<10 cigs (moderate)	76 (14.4%)	63 (14.0%)	13 (17.8%)	0.61 (0.31–1.23)	0.173
>10 cigs (intense)	99 (18.9%)	84 (18.6%)	15 (20.6%)	0.71 (0.37–1.37)	0.311
Missing	32 (6.1%)	23 (5.1%)	9 (12.3%)	Not included	
BMI (kg/m^2^) (SD)	24.7 (±4.7)	24.7 (±4.7)	24.8 (±4.7)	0.99 (0.94–1.05)	0.891
Waist circompherence (cm) (SD)	89.1 (±12)	89.2 (±12)	88.6 (±12)	1.08 (0.98–1.03)	0.467
Lipodystrophy					
No lipodystrophy	184 (35.0%)	144 (31.9%)	40 (54.8%)	1 (ref.)	–
Lipoatrophy	129 (24.6%)	112 (25.0%)	16 (21.9%)	1.96 (1.04–3.68)	0.036
Fat accumulation	212 (40.4%)	195 (43.1%)	17 (23.3%)	3.18 (1.73–5.84)	<0.001
Cardiovascular and metabolic					
Triglycerides (mg/dL) (medican, IQR)	142 (95–207)	143 (96–209)	127 (93–185)	1.00 (0.99–1.00)	0.494
Total cholesterol (mg/dL) (mean, SD)	198 (±45)	197 (±45)	197 (±43)	1.00 (0.99–1.00)	0.929
HDL (mg/dL) (mean, SD)	46 (±14)	46 (±14)	47 (±16)	0.99 (0.98–1.00)	0.645
LDL (mg/dL) (mean, SD)	117 (±33)	117 (±33)	119 (±33)	0.99 (0.99–1.00)	0.710
Glucose (mg/dL) (mean, SD)	94 (±19)	95 (±21)	90 (±10)	1.03 (1.01–1.05)	0.011
HOMA (median, IQR)	2.4 (1.4–3.8)	1.9 (1.2–2.9)	1.9 (1.2–3.0)	1.29 (1.09–1.54)	0.003

**Fig. 1:**
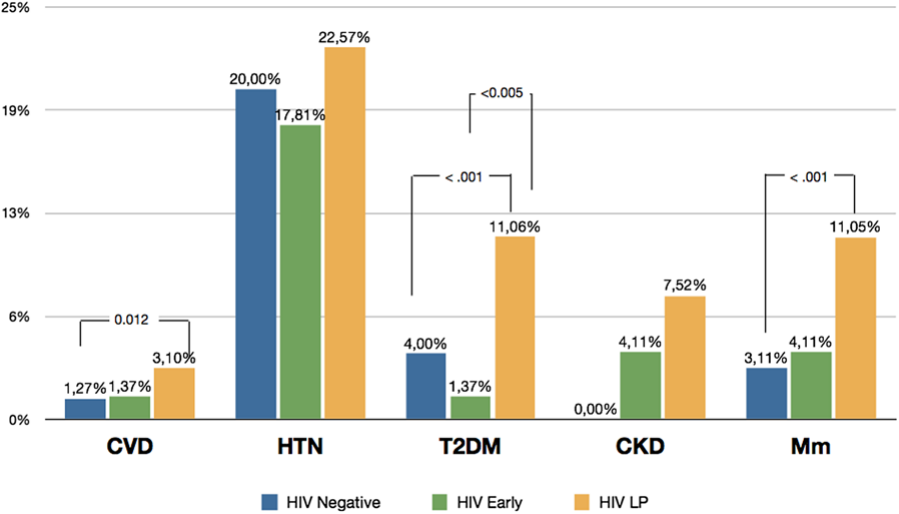
NICM and MM prevalence distribution across study groups

NICM prevalence are reported in Fig. 1. A trend in higher prevalence of any NICM was observed in HIV + LP participants. With regard to MM the difference in prevalence reached significant levels when comparing HIV- and HIV + EP with HIV + LP (respectively p < 0.001 and p < 0.001), being present in 11% in HIV + LP, 4% in HIV + EP and 3% in HIV-negative controls, respectively.

**Fig. 2:**
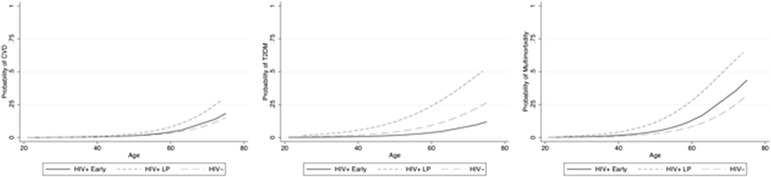
Age-related risk probability for CVD, T2DM and MM

According to our study hypothesis we explored the probability of NICM and MM across age distribution using a logistic model in the three comparative groups. CVD, T2DM and MM were significantly higher in HIV + LP when compared to both HIV + EP and HIV-negative participants (p < 0.01 for all comparisons) (Fig. 2).

No difference was found in hypertension risk across age distribution in the three groups.

CKD and low BMD data were not available for comparison in the HIV-negative group, but a non-significant trend in higher prevalence was observed in HIV + LP when compared to HIV + EP, respectively 7.52 vs. 4.11%, p = 0.291 and 20.13 vs. 12.33%, p = 0.147.

**Fig. 3:**
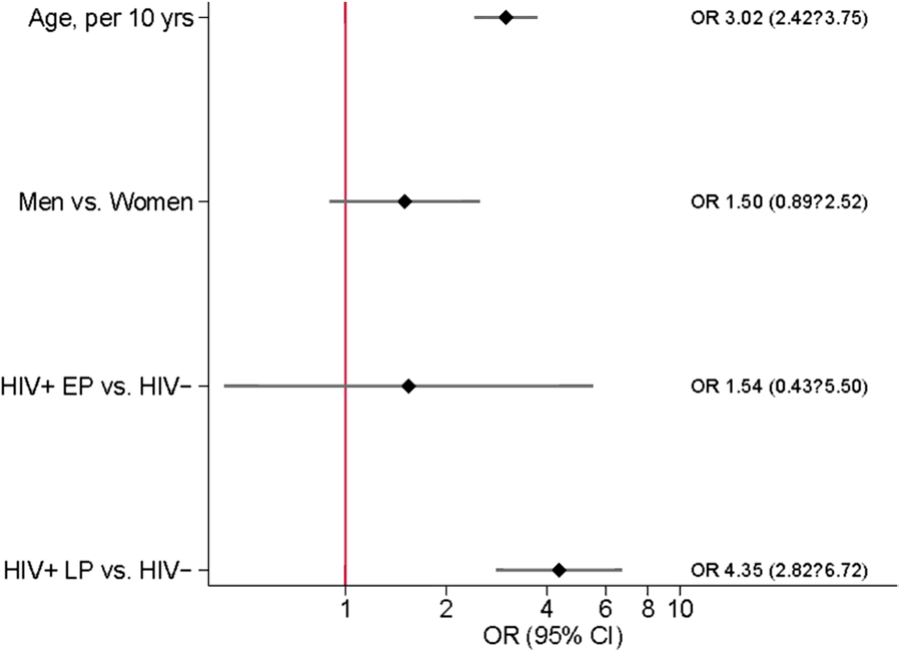
Multivariable logistic regression model to detect independent predictors of MM in the cohort

In multivariable models including age and sex, we observed fourfold increased odds of MM in HIV + LP participants compared to HIV-negative individuals (OR 4.3, 95% CI 2.8–6.7, p < 0.001). On the contrary, HIV + EP was not a risk factor for MM when compared to HIV-negative (OR 1.5, 95% CI 0.4–5.5, p = 0.503) (Fig. 3).

**Fig. 4:**
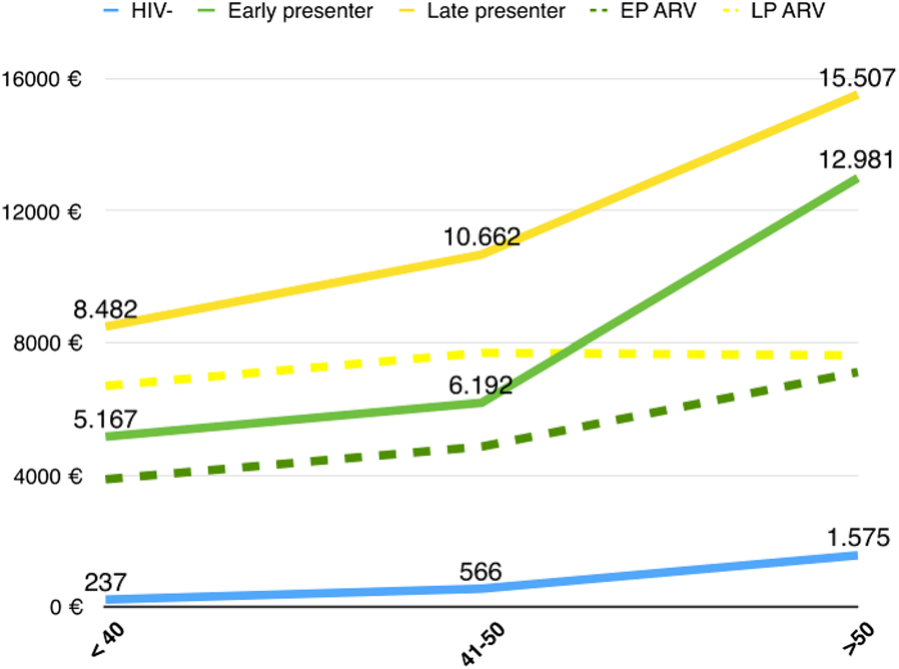
Total and cART costs of medical care in HIV negative, HIV + EP and HIV + LP across age strata (mean)

With regards to our secondary objective we estimated NICM related costs, HIV outpatient costs and cART costs in the 3 patient groups. Additional file 1: Figure S1 describes total costs across age strata and Fig. 4 also stratify HIV patients in EP and LP.

A multivariable logistic regression was built to predict total cost in the whole population including clinically significant covariates (Table 2).

**Table 2:** Independent predictors of total cost in the whole population

	β-coeff	95% CI	p value
HIV + EP vs. HIV−	1400	422–2380	0.005
HIV + LP vs. HIV−	3246	2233–4260	<0.001
Multimorbidity	6578	6049–7107	<0.001
Age	53	41–64	<0.001
Sex			
Women	0 (Ref)	–	–
Men	114	−106 to 334	0.309
ART	5606	4602–6610	<0.001

A second linear regression model was built to identify independent predictors of total cost in HIV-infected patients only. Predictors of total cost in cases were: LP (β, 2994; CI 1561–4427, p < 0.001); MM (β, 7818; CI 5653–9985, p < 0.001); age (β, 150; CI 87–212, p < 0.001); current CD4 count < 200 (β, 2723; CI −270 to 5716, p = 0.075); male sex (β, 281; CI −865 to 1427, p = 0.630); protease inhibitors (PIs) exposure in months (β, 33; CI 6–60, p = 0.015).

## Discussion

Data from MHMC cohort study show that individuals with late presentation for HIV diagnosis (HIV + LP) exhibit an increased risk for age-related NICM and MM compared to individuals with early presentation for HIV diagnosis (HIV + EP) and matched HIV-negative individuals sampled from the general population. This difference translates into increased total costs of medical care in all age strata, but appear particularly relevant in patients above the age of 50. Therefore, LP influences both clinical and economical outcomes.

At any age, the risk for individual NICM as well as MM was fourfold higher in HIV + LP compared to people without HIV, while HIV + EP had an intermediate risk.

Our results should be interpreted with caution, in particular with regard to reproducibility of our results in different HIV settings. It could be argued that the tertiary referral setting of the MHMC may concentrate patients with higher prevalence of NICM and MM, however the prevalence of these conditions has previously been demonstrated to be similar between local MHMC attendees and those referred from other centers [18]. In Italy, people with HIV have full free access not only to cART but also to clinical care including diagnostic procedures with no co-pay. This might result in a selection bias as screening activities are offered relatively more frequently to individuals with HIV than the general population, which might result in higher rates of incidentally identified asymptomatic disease. The information available in the CINECA-ARNO administrative database limited further comparisons in rates of NICM diagnoses, as not all major risk factors for NICM are collected and clinical assessments are not provided. Moreover, with regard to CKD, the number of clinical events was very limited and BMD measurements were not available in CINECA cohort. Furthermore, the HIV + EP group is relatively small. Finally, the cross-sectional design of our study cannot prove causality regarding the impact of LP on NICM or MM risk, nor we were able to collect biomarkers of systemic and tissue inflammation to argue a pathogenic link between LP and these outcomes.

The total cost of medical care was higher in HIV-positive participants than in the general population in any age strata. Higher costs was, as expected, mainly attributable to antiretroviral drugs costs, especially PIs [27–29]. Of interest, total and ART costs for the HIV + EP group aged less than 40 years is much lower than the other HIV + EP strata. This can be explained with the fact that 30.3% of these patients were not on ART, following WHO 2013 guidelines [30].

Notably NICM-related care costs contributed significantly to the higher cost burden of HIV-infected patients in the context of LP.

These findings reinforce what has already been suggested by Krentz and Gill, that early initiation of ART may result in a cost-saving intervention [24]. Conversely, increasing age and concurrent MM represent a good indicator of increased total cost.

We believe that, in a demographic setting, particularly in a resource-rich setting, in which the majority of HIV-infected patients are older than 50 years, total care cost will continue to increase [28, 31, 32].

These results suggest that, in the absence of targeted HIV prevention and diagnostic campaigns, the proportion of HIV+/LP will continue to increase, which will increase the burden of higher MM-related clinical and economic costs. In light of the unmistakable message recently provided by the strategic timing of antiretroviral therapy (START) study [33], which demonstrated a beneficial effect of immediate antiretroviral therapy for both serious AIDS-related and serious non–AIDS-related events, we can hope a reduction of the proportion of HIV + LP with a reduction in the burden and cost of comorbidities.

In conclusion, LP with HIV infection remains very common today in Italy and is associated with higher NICM and MM which is associated with higher total care costs. Promoting early testing and access to care is still urgently needed.

## Additional file

**Additional file 1: Table S1.** Reference Costs according to ICD9 codes and HIV direct costs. Figure S1. Total costs in HIV negative, Early and Late Presenters by age. Figure S2. NICM cost in HIV negative, early and late presenters by age.

## Abbreviations

AIDS: acquired immuno deficiency syndrome
AIFA: Agenzia Italiana del Farmaco
BMD: bone mineral density
cART: combined anti retroviral therapy
CKD: chronic kidney disease
COHERE: collaboration of observational HIV epidemiological research Europe
CVD: cardio vascular disease
DXA: dual-energy X-ray absorptiometry
EP: early presentation
HIV: human immunodeficiency virus
HIV + EP: HIV-positive patients with early presentation
HIV + LP: HIV-positive patients with late presentation
ICD-9: international classification of diseases, ninth edition
IRIS: immune reconstitution inflammatory syndrome
LP: late presentation
MDRD: modification of diet in renal disease
MHMC: Modena HIV metabolic clinic
MM: multi morbidity
NICM: non infectious comorbidities
START: strategic timing of antiretroviral therapy
T2DM: type 2 diabetes mellitus
