# Supplementary material for: Late presentation increases risk and costs of non-infectious comorbidities in people with HIV: an Italian cost impact study
Source: AIDS Res Ther. 2017 Feb 16;14:8. doi: 10.1186/s12981-016-0129-4 (PMC5311843; doi:10.1186/s12981-016-0129-4)

**Supporting data**

**Additional file 1: Table S1. Reference Costs according to ICD9 codes and HIV direct costs.**

| Pathology | Mean cost |
| --- | --- |
| CVD | € 7238.49 |
| Revascularization | € 8506.73 |
| Renal Failure | € 16186.50 |
| Bone fracture | € 3724.71 |
| Hypertension | € 1922.05 |
| Diabetes Mellitus | € 2296.28 |
| HIV  <75 CD4  76-200 CD4  >201 CD4 | € 1066.00  € 958.00  € 147.00 |

**Additional file 1: Figure S1. Total costs in HIV negative, Early and Late Presenters by age.** Supplementary figure 1 reproduces total costs distribution in the 3 groups (HIV+EP, HIV+LP and HIV-) divided by age.


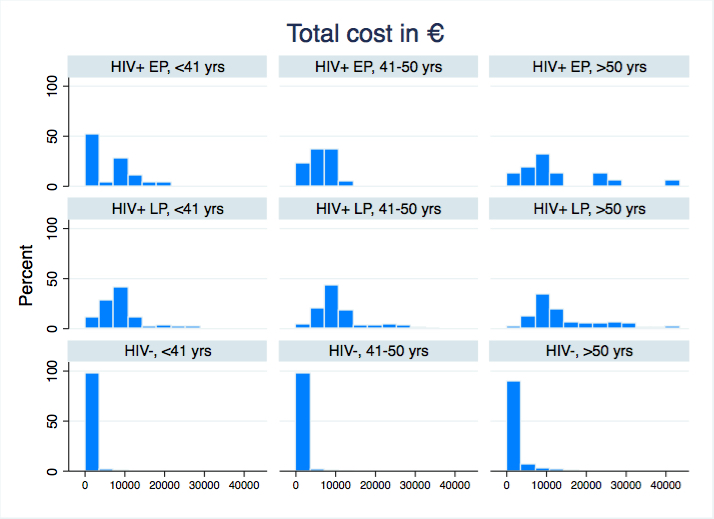


**Additional file 1: Figure 2. NICM cost in HIV negative, Early and Late Presenters by age.** Supplementary figure 1 reproduces costs distribution excluding cART to underline the contribution of NICM costs in patients above the age of 50 years.


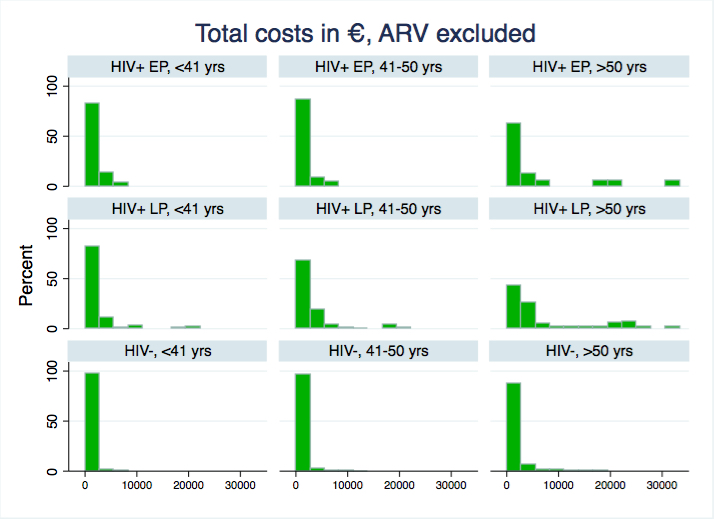

Supplement: Supplementary file 1 — Additional file 1: Table S1. Reference Costs according to ICD9 codes and HIV direct costs. Figure S1. Total costs in HIV negative, Early and Late Presenters by age. Figure S2. NICM cost in HIV negative, early and late presenters by age. [file 12981_2016_129_MOESM1_ESM.docx]
